# Integrated care for older people (ICOPE) implementation framework, 2nd edition: getting closer to the medicine of the twenty-first century

**DOI:** 10.1007/s41999-026-01420-2

**Published:** 2026-02-12

**Authors:** M. Cristina Polidori, Dorota Religa, Jerzy Gasowski, Esa Jämsen, Finbarr C. Martin

**Affiliations:** 1 Ageing Clinical Research, Department II of Internal Medicine and Center for Molecular Medicine Cologne, University of Cologne, Faculty of Medicine and University Hospital Cologne, Cologne, Germany; 2 Cologne Excellence Cluster on Aging and Aging Associated Diseases (CECAD), University of Cologne, Cologne, Germany; 3 Division for Clinical Geriatrics, Department of Neurobiology, Care Sciences and Society (NVS), Karolinska Institute, Stockholm, Sweden; 4 Department of Internal Medicine and Gerontology, Jagiellonian University Medical College, Kraków, Poland; 5 Faculty of Medicine (Clinicum), University of Helsinki, Helsinki, Finland; 6 Population Health Sciences, King’s College London, London, UK

We are now halfway through the United Nations Decade of Healthy Ageing and the WHO continue in its key role in galvanizing global change towards more ageing-attuned healthcare. Here, we review the second edition of the *Integrated Care for Older People* (*ICOPE*) guideline released by WHO in late 2024 [1], and now available in several languages and as an App. This guidance marks a significant evolution in global strategies to support healthy ageing amid rising longevity. This updated handbook refines the framework for person-centered assessment and care pathways, drawing on real-world implementation experiences to better equip health systems worldwide. To contextualize this advancement, the ICOPE guideline aligns seamlessly with the WHO's broader framework on healthy ageing, as outlined in the World Report on Ageing and Health [2]. As a deliberate move away from a disease-focused approach, the Report offered a new definition of health ageing: “*the process of developing and maintaining the functional ability that enables wellbeing in older age*.” Functional ability encompasses what individuals can do and be in their environments, determined by the interplay between intrinsic capacity—the composite of all physical and mental capacities an individual can draw upon—and their environment with all its social–cultural and physical dimensions. This framework shifts focus from curative models to proactive maintenance of intrinsic capacity, addressing the demographic shift, where older populations are expanding rapidly, particularly in low- and middle-income countries (LMICs).

**Fig. 1:**
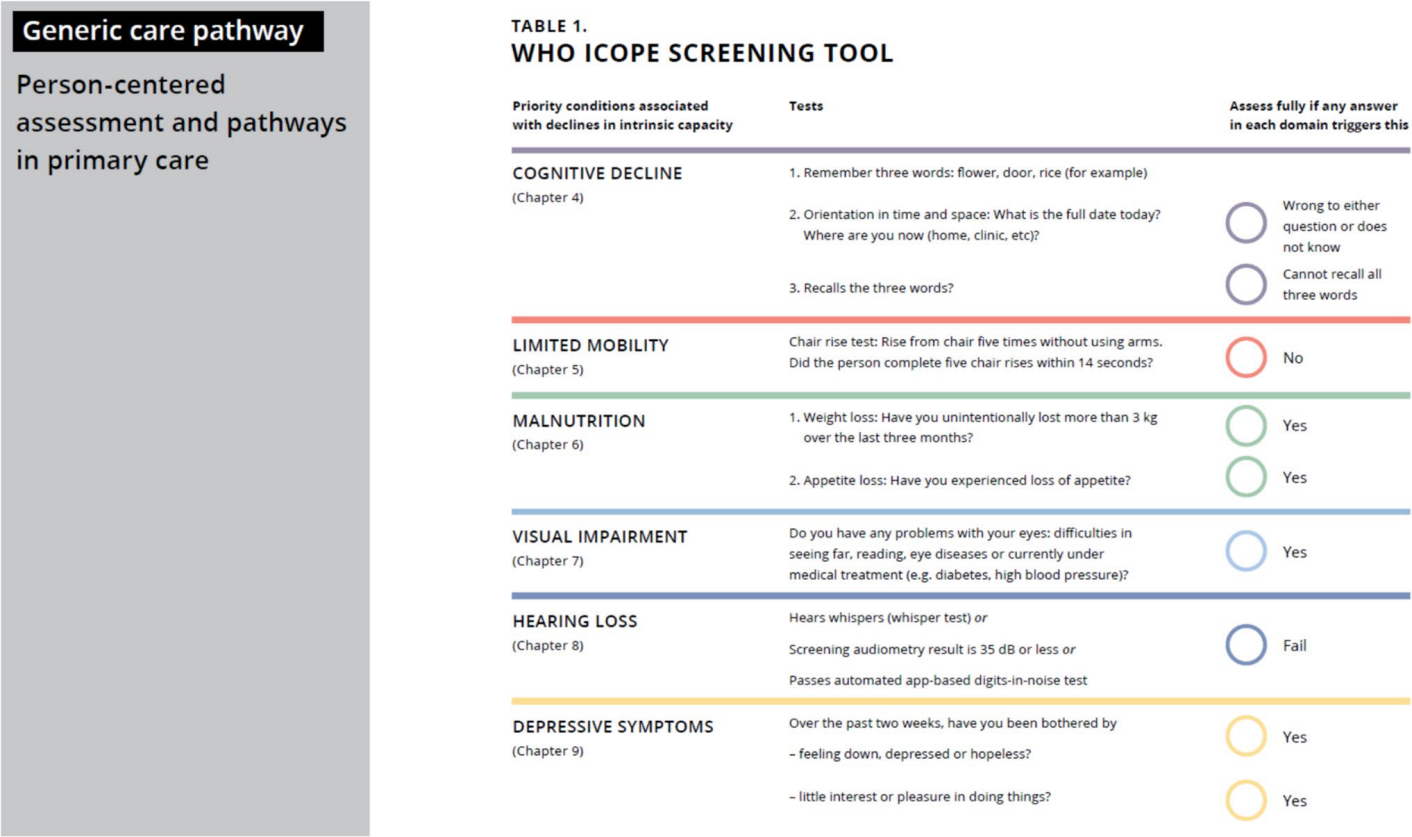
Basic assessment for loss of intrinsic capacity, which usually takes 8–12 min, can be conducted by trained community health workers, community volunteers, nurse assistants, nurses, doctors and social workers or other health workers. It is strongly recommended to assess all domains of intrinsic capacity at one time, not separately. For cognition, vision and hearing, a filter question is suggested. If the older person’s response is YES, they can be referred directly for an in-depth assessment (Step 2) and no further basic assessment for these domains is necessary. The basic assessment can be adapted to the local health system and capacity of health workers

The structure of intrinsic capacity domains in the ICOPE guideline is rigorously justified by foundational research, emphasizing a multidimensional view of ageing (Fig. 1). Novel analysis of epidemiological data identified five core domains—locomotion, vitality, cognition, psychological, and sensory (vision and hearing)—as essential for capturing an individual's reserves and potential for healthy ageing [3]. These domains provide a comprehensive metric beyond traditional disease-focused assessments, allowing for early detection of declines that could lead to disability. This domain-based approach validates ICOPE's emphasis on preventive interventions, as it enables targeted strategies to bolster capacities before irreversible losses occur, fostering resilience in ageing populations. Complementing this, reframing healthy ageing around functional ability underscores the importance of age-friendly environments which requires combating ageism as well as developments in design and technologies [4].

At its core, the ICOPE guidance pathway is structured to enable high-quality care, explicitly designed for primary and community care settings, where resources may be limited. It unfolds in four practical steps: (1) a basic assessment to identify declines in intrinsic capacity, plus provision of generic health promotional advice; (2) an in-depth evaluation for those flagged; (3) the development of a personalized care plan involving multidisciplinary input; and (4) ongoing care plan monitoring with specialist referrals as needed. This pathway empowers general practitioners but also trained non-experts, such as community health workers or nurses, to implement it without requiring specialized geriatric expertise, making it accessible in diverse contexts from urban clinics to rural outreach programs. By prioritizing person-centered goals—what matters most to the individual—it integrates assessments of mobility, nutrition, sensory functions, cognition, mood, and vitality into actionable plans that promote functional ability, independence and reduce unnecessary hospitalizations.

Compared to the inaugural 2019 edition, the 2024 ICOPE handbook introduces several enhancements that reflect lessons from global pilots and evolving evidence. Notably, it expands the scope to include more detailed guidance on integrating digital tools for monitoring, such as mobile apps for self-assessment, which improve scalability and user engagement. The updated version also strengthens emphasis on environmental adaptations and social support, addressing gaps in the original by incorporating feedback from implementations that highlighted the need for better linkage between assessments and community resources. In addition, it refines screening thresholds and care algorithms based on data from diverse populations, potentially reducing false positives and enhancing precision in identifying at-risk individuals. These improvements could lead to more efficient resource allocation, lower healthcare costs, and better outcomes by preventing frailty progression earlier, particularly in settings with high multimorbidity burdens.

Where does this approach with intrinsic capacity fit into the historical development of healthcare systems? Complexity of ageing medicine and geriatrics arise from the interplay of biological changes, multimorbidity, medication management challenges, lifestyle and the need for comprehensive, individualized care strategies. The 1948 WHO definition of health as a “*complete state of physical, mental and social wellbeing*” acknowledged the biopsychosocial nature of health and its determinants [5] and is consistent with the health and wellbeing unit of the UN sustainable goals [6]. However, the definition also included “…*and not merely the absence of disease or infirmity*.” In contrast, the healthy ageing framework in the 2015 WHO Aging Report proposed a new definition: “*healthy ageing is the process of developing and maintaining the functional ability that enables wellbeing in older age*”. This reorientation towards functional ability and away from disease and its absence was built on the insights underpinning the International Classification of Functioning, Disability and Health [7]. The WHO framework explains functional ability as arising from the interaction between an individual’s intrinsic capacity and her/his environment in all its physical, social and cultural dimensions. Importantly, this perspective resonates with the overarching aim and the interventions incorporated into comprehensive geriatric assessment (CGA) [8].

This does not negate the need to understand the biological processes involved in ageing and age-related conditions, as working with the traditional biological model of disease has enabled the doubling of life expectancy since the past century, but multimorbidity and fragmented care, frequent medical visits and high medication burden are increasing the risks of disability and hospitalizations due to drug–drug interactions, drug–disease interactions, and adverse drug events. The limitations of the biological approach to disease have, therefore, come into question given the rapidly increasing number of older and chronically ill patients, and the developments of the past 20 years towards biopsychosocial strategies.

The role of the ICOPE approach, centered on intrinsic capacity, is still emerging, yet it has shown promising traction through implementations in several LMICs and at a national scale in France. Pilot programs in countries such as India, Mexico, and Vietnam have demonstrated feasibility in resource-constrained environments, where community health workers use simplified screening tools to identify and manage declines, leading to improved functional outcomes and reduced healthcare utilization [9]. In France, the INSPIRE ICOPE–CARE program in the Occitania region has scaled up dramatically, enrolling thousands of seniors since 2020 and integrating digital monitoring with primary care pathways to prevent disability [10]. By October 2024, over 3000 individuals were actively followed, with high adherence rates and evidence of delayed functional loss, underscoring the model's adaptability and potential for widespread adoption. These efforts highlight ICOPE's transformative potential, bridging historical biopsychosocial ideals with modern, scalable interventions to redefine ageing as an opportunity rather than a burden.

As global populations age, the 2024 ICOPE edition arrives at a pivotal moment, offering a blueprint for equitable, proactive care. Yet, its success hinges on broader adoption, investment in training, and ongoing research to refine its impact across cultures and economies. Most of the health care provided will be by general practitioners and colleagues in community care, but the geriatrician plays a pivotal role by serving as the specialist resource to an integrated system. This can involve sharing knowledge and experience, providing more complex assessments and direct care to the more frail older people and contributing to the clinical development and governance of the care pathways. By embedding intrinsic capacity into routine practice, we can aspire to a future, where older adults not only live longer but thrive with dignity and purpose. This is more than an update—it is a call to action for health systems worldwide to embrace a truly integrated vision of ageing.
